# An NMR crystallography investigation of furosemide

**DOI:** 10.1002/mrc.4789

**Published:** 2018-10-11

**Authors:** Miri Zilka, Jonathan R. Yates, Steven P. Brown

**Affiliations:** ^1^ Department of Physics University of Warwick Coventry United Kingdom; ^2^ Department of Materials University of Oxford Oxford United Kingdom

**Keywords:** NMR crystallography, solid‐state NMR

## Abstract

This paper presents an NMR crystallography study of three polymorphs of furosemide. Experimental magic‐angle spinning (MAS) solid‐state NMR spectra are reported for form I of furosemide, and these are assigned using density‐functional theory (DFT)‐based gauge‐including projector augmented wave (GIPAW) calculations. Focusing on the three known polymorphs, we examine the changes to the NMR parameters due to crystal packing effects. We use a recently developed formalism to visualise which regions are responsible for the chemical shielding of particular sites and hence understand the variation in NMR parameters between the three polymorphs.

## 1 INTRODUCTION

A definition for polymorphism was given by McCrone in 1965: a polymorph is a solid crystalline phase of a given compound resulting from the possibility of at least two different arrangements of the molecules of that compound in the solid state.^1^ Polymorphism is a common phenomena, with one in three compounds in the Cambridge Structural Database (CSD) exhibiting polymorphism.^2^ When several polymorphs exist, they will often exhibit different macroscopic properties despite having the same molecular composition. Variations can be observed in, for example, stability, strength, and elasticity.^3^ For the pharmaceutical industry, the bioavailability of a compound is of particular importance since it can determine if a drug can be administrated as a tablet or not. An active pharmaceutical ingredient can have a low solubility in water in one form and a more desirable solubility in another form or a cocrystal.

The key to a crystal's macroscopic characteristics is found in its microscopic packing arrangement. Although composed of the same molecular units, the molecules can interact with each other differently resulting in different properties. The polymorphs can vary in unit‐cell dimensions, symmetries, intermolecular bonds, stacking arrangements, and conformational changes in the molecular unit itself.

Solid‐state nuclear magnetic resonance (NMR) is a highly sensitive probe of the local atomic environment, making it an effective tool for distinguishing between similar polymorphs.^10^, ^11^, ^12^ Under the umbrella of NMR crystallography,^13^, ^14^, ^15^ solid‐state NMR experiments can be used in conjunction with first‐principles calculations, for example using the gauge‐including projector augmented wave (GIPAW) approach,^16^, ^17^ to validate structures solved by powder X‐Ray diffraction (pXRD). Solid‐state NMR, being a sensitive local probe,^10^, ^18^, ^19^, ^20^, ^21^ has a complementary nature to pXRD that relies on long‐range order. With first‐principles GIPAW calculations tying these two techniques together, NMR crystallography has a proven track record in analysing and validating solid structures.^22^, ^23^, ^24^, ^25^, ^26^, ^27^, ^28^

The influence of intermolecular effects on NMR chemical shifts can be studied by comparing the calculated chemical shifts from an isolated molecule extracted from the crystal structure to the calculated chemical shifts for the full crystal structure.^18^, ^20^, ^29^, ^30^ This difference reflects two contributions: long‐range effects of current elements, for example, ring currents, and local changes in electronic structure that result from crystal packing, for example, hydrogen bonding. The nuclear independent chemical shift (NICS)^31^, ^32^ can be used to identify aromatic ring currents that can have a strong effect on the ^1^H solid‐state NMR chemical shift.^30^, ^33^

**Scheme 1:**
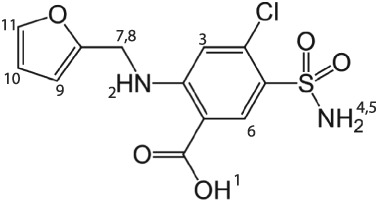


**Figure 1:**
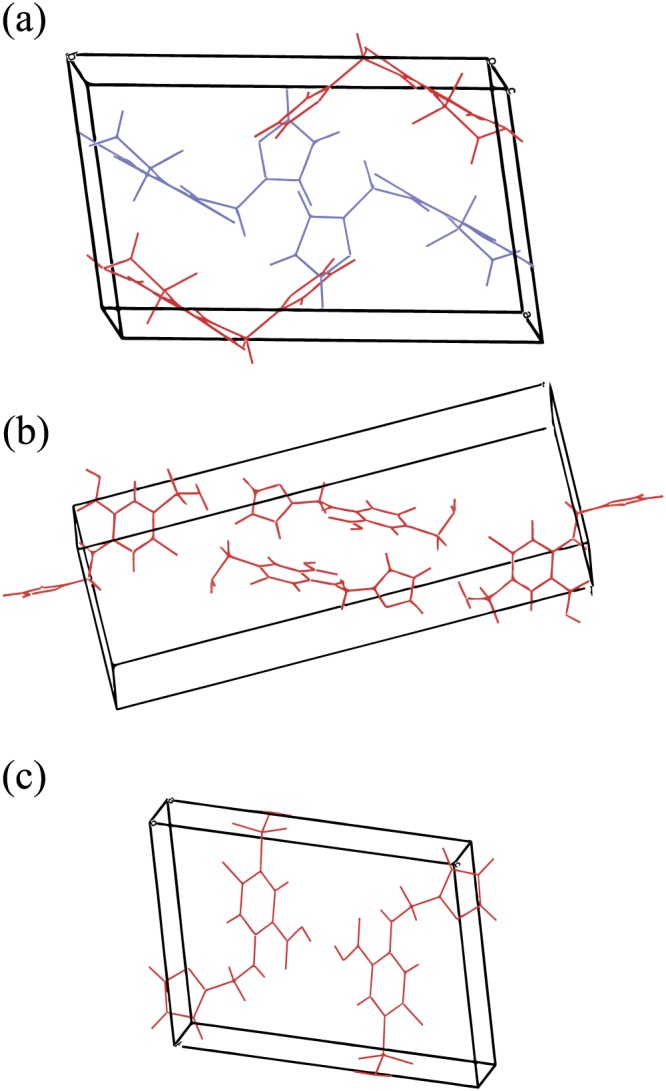
Crystal structures for furosemide polymorphs. Same coloured molecules are related by symmetry. (a) Form I (FURSEM01), (b) Form II (FURSEM14), and (c) Form III (FURSEM16)

Furosemide (Scheme 1) is an active pharmaceutical ingredient marketed under the brand name Lasix. Furosemide is used to relieve congested fluids in partly or fully failing organs such as the heart, liver, and kidney^34^ and is also used to treat hypertension.^35^ Furosemide has three known forms, with 10 entries in the CSD. See Table 1 for a summary of all entries and Figure 1 for representations of the crystal structures of the three forms. The correct form^36^ of form I is known to have *Z* *′*  =  2 and *Z*  =  4, and a recent study using GIPAW calculations that considered FURSEM01, FURSEM17, and a new form and solid‐state NMR experiments has determined that FURSEM17 is likely an inaccurate solution of form I.^9^ Unfortunately, furosemide has poor bioavailability^37^, ^38^, ^39^ and furosemide cocrystals^40^, ^41^, ^42^ have been synthesised in an attempt to improve the solubility in water. Furosemide and its cocrystals have been previously studied using solid‐state NMR.^42^, ^43^, ^44^, ^45^, ^46^, ^47^ Dissolution kinetics of furosemide form I have also recently been studied using in situ 3D microscopy.^48^

**Table 1:** CSD entries for furosemide

Form I	$P\bar{1}$	*Z* *′* = 2	FURSEM^4^	**FURSEM01** 5
			FURSEM02^5^	**FURSEM03** 6
			**FURSEM13** 7	FURSEM17^8^
			FURSEM18^9^
Form II	*P*2_1_/*n*	*Z* *′* = 1	**FURSEM14** 7	FURSEM15^7^
Form III	$P\bar{1}$	*Z* *′* = 1		**FURSEM16** 7

*Note*. FURSEM02 has *Z* *′*  =  1. CSD: Cambridge Structural Database.

In this study, we analyse three solutions for form I: FURSEM01 (F01),^5^ FURSEM03 (F03),^6^ and FURSEM13 (F13)^7^ and one solution for each of forms II and III, FURSEM14 (F14)^7^ and FURSEM16 (F16),^7^ respectively. For form I of furosemide, the calculated values are compared with solid‐state NMR experimental spectra obtained using ^13^C CP magic‐angle spinning (MAS), ^1^H–^13^C refocused INEPT, and ^1^H double quantum (DQ) MAS experiments. The GIPAW‐calculated NMR chemical shifts are analysed focusing on intermolecular hydrogen bonding and aromatic ring current effects. The analysis notes subtleties between solved structures of the same form, and different geometry optimization approaches.

## 2 EXPERIMENTAL AND COMPUTATIONAL DETAILS

### 2.1 Experimental details

Furosemide was purchased from Sigma‐Aldrich (Gillingham, UK). Solid‐state NMR experiments were performed using Bruker Avance III NMR spectrometers operating at a ^1^H Larmor frequency of 500.1 MHz (^13^C Larmor frequency of 125.8 MHz). A Bruker 4‐mm triple resonance MAS probe (in double‐resonance mode) was used in ^1^H–^13^C CP MAS and refocused INEPT experiments, and a Bruker 2.5‐mm double resonance MAS probe was used for the ^1^H DQ experiment. In ^1^H–^13^C CP MAS and refocused INEPT experiments, SPINAL64 ^1^H heteronuclear decoupling^49^ with a pulse duration of 2.5 μs was applied for an acquisition time of 40 ms. The ^1^H nutation frequency for pulses and decoupling was 100 kHz.

A pulse sequence and coherence transfer pathway diagram for the ^1^H (SQ‐DUMBO)–^13^C SQ refocused INEPT experiment can be found in Fig. 5 of Elena et al.,^50^ and for ^1^H DQ MAS,^51^ using BABA (back‐to‐back) recoupling^52^, ^53^ in Fig. 7 of Brown and Spiess.^54^ For the ^1^H DQ MAS experiment, a 16‐step phase cycle was used to select Δp  =  ±2 on the DQ excitation pulses (four steps) and Δp  =  ±1 (four steps) on the z‐filter 90° pulse, where p is the coherence order. For the 2D ^1^H–^13^C refocused INEPT experiment, eDUMBO‐1_22_ homonuclear decoupling,^55^, ^56^ was employed during the ^1^H evolution period and the spin‐echo durations. The 32‐μs eDUMBO‐1_22_ cycle was divided into 320 steps of 100 ns. The STATES‐TPPI method was used to achieve sign determination in F_1_ in the refocused INEPT and DQ MAS experiments.

### 2.2 Computational details

Calculations were performed using a developer version of the CASTEP code^57^ together with the CASTEP9 set of ultrasoft pseudopotentials.^58^ The cut‐off for the basis set was 800 eV, and the Brillouin Zone was sampled using a Monkhorst Pack^59^ grid with a minimum spacing of 0.05 × 2π Å^−1^. All crystal structures were optimised using the Perdew‐Burke‐Ernzerhof (PBE)^60^ functional together with dispersion corrections using the Tkatchenko‐Scheffler (TS) scheme.^61^ Two separate geometry optimisations were performed for each structure: In the first case (denoted “fixed cell”), only the internal coordinates were allowed to change, with the unit cell dimensions fixed to the X‐ray diffraction derived values. In the second case (denoted “relaxed cell”), both unit cell parameters and internal coordinates were able to relax during the optimisation. Calculations of NMR magnetic shieldings were computed using the GIPAW approach.^16^, ^17^ All crystal structures and full shielding tensors for all atoms are made available as a downloadable data set. Structure views for Figure 6 were generated using VESTA 3^62^; structure views for Figure 1 and root‐mean‐square deviation of atomic positions were generated using Mercury CSD.^63^

### 2.3 Molecule to crystal change in magnetic shielding and NICS

Isotropic chemical shifts, δ_iso_, are related to the calculated isotropic magnetic shieldings, σ_iso_, by a reference value σ_ref_ (all in ppm):

(1) $$\delta_{iso}=\sigma_{ref}-\sigma_{iso}.$$

The necessary magnetic shielding calculations to compute the molecule to crystal change in chemical shift and the NICS are illustrated in Fig. 1 of Zilka et al.^64^ The first calculation (denoted I_fullcell_) is of a single crystallographic unit cell. The second calculation (denoted I_nomol_) is performed on a unit cell of the crystal with the molecule that contains the atom of interest removed. Depending on the crystal structure, it may be necessary to simulate a supercell of the unit cell, such that the missing molecule is surrounded by all of its nearest neighbours. In this work, a 2 × 1 × 1 supercell was used for the NICS calculations of all three polymorphs. *
FURSEM01 does not employ the convention α  <  β  <  γ, and α and γ are swapped. To account for this, a 1 × 1 × 2 supercell was used.The NICS, σ_NICS_, is obtained from calculating the value of the magnetic shielding at the site of the atom in the missing molecule. The final calculation (denoted I I I_onemol_) is a vacuum supercell with only one molecule containing the atom of interest. The molecule to crystal change in chemical shifts Δδ is the change in shifts between calculations I I I_onemol_ and I_fullcell_. Within this setup, we can analyse all intermolecular effects. The values denoted as “H bond strength” correspond to the sum of the molecule to crystal change in chemical shift and the NICS; as such, this accounts for changes in the NMR chemical shift due to interactions between a specific molecule and its neighbours.

## 3 RESULTS

### 3.1 Form I of furosemide

**Figure 2:**
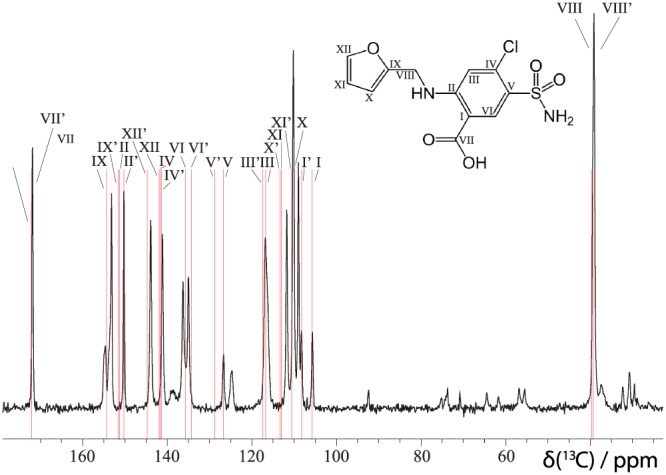
A ^1^H (500 MHz)^-13^C CP (2 ms contact time, 800 transients were co‐added for a recycle delay of 105 s) MAS (10 kHz) NMR spectrum of furosemide (form I) (black) with the GIPAW calculated ^13^C chemical shifts for FURSEM01 overlaid in red

**Figure 3:**
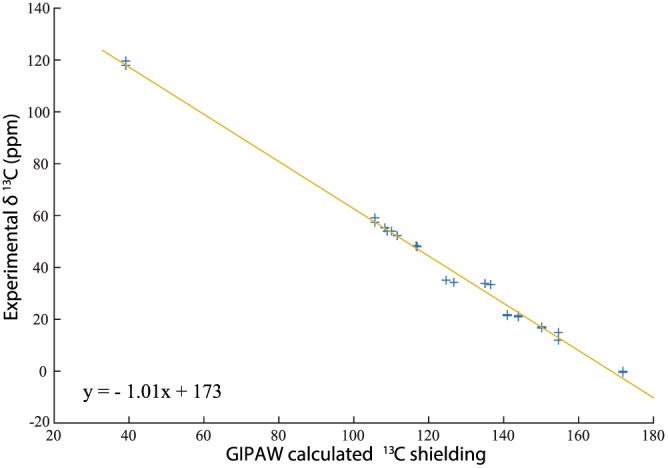
Plot of the experimental ^13^C chemical shifts of furosemide (form I) against the GIPAW calculated absolute isotropic ^13^C shielding for the 12 carbon atoms in the two distinct furosemide molecules in the asymmetric unit cell (FURSEM01). The line of best fit had not been constrained to a gradient equal to −1

A ^13^C CP MAS spectrum of furosemide form I, recorded at room temperature, is presented in Figure 2. Furosemide form I contains two molecules in the asymmetric unit (Z ′  =  2), each with 12 carbon atoms, corresponding to, at most, 24 peaks in the ^13^C spectrum. The ^13^C CP MAS spectrum is consistent with previously reported data.^45^ The spectrum was assigned using the GIPAW calculated chemical shifts from the geometry optimised (relaxed) FURSEM01 unit cell. The fit between the experimental spectrum and the calculated peaks is very good with the exception of the carbon atoms closest to the sulfur atom (V ′). Moreover, the splitting in the values of the chemical shift between the two inequivalent molecules is also well reproduced. In Figure 3, the experimental ^13^C chemical shifts are plotted against the GIPAW‐calculated absolute isotropic ^13^C chemical shielding of furosemide form I. The (negative) gradient (see Equation 1) was allowed to deviate from unity. It is common practice to also fit the data while constraining the gradient to unity^28^; however, with the unconstrained slope being 1.01, no additional fit was done. The intercept with the y‐axis usually determines the reference shielding, σ_ref_, used to relate calculated and experimental chemical shifts.

**Figure 4:**
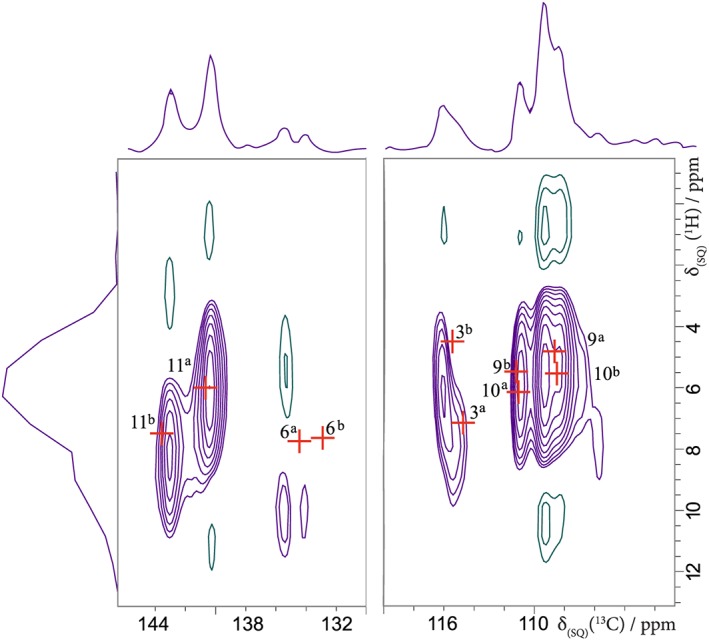
A ^1^H (500 MHz)‐^13^C refocused INEPT (eDUMBO‐1_22_^1^H homonuclear decoupling) MAS (12.5 kHz) NMR spectrum, together with skyline projections, of furosemide form I, recorded using a spin‐echo (τ – π – τ) duration, τ  =  τ ^′^  =  0.96 ms. Two hundred and fifty‐six transients were coadded for each of 26 t_1_ FIDs for a recycle delay of 60 s, corresponding to a total experimental time of 4 days and 15 hr. The base contour level is at 15%. Crosses (in red) correspond to the GIPAW calculated ^13^C and ^1^H chemical shifts for directly bonded CH moieties, using σ_ref_  =  29.6 ppm for ^1^H and σ_ref_  =  168.4 ppm for ^13^C for the geometry optimised (CASTEP) crystal structure of furosemide form I based on CSD structure FURSEM01

A 2D ^1^H–^13^C refocused INEPT^50^ spectrum is shown in Figure 4. The spectrum was recorded using moderate 12.5‐kHz MAS, with eDUMBO‐1_22_^1^H homonuclear decoupling, with short spin‐echo durations selective for the observation of direct one bond CH connectivities. The experimental spectrum is overlaid with the GIPAW calculated ^1^H and ^13^C chemical shifts. Because only a small region of the full ^13^C chemical shift range is presented in Figure 4, a small variation from the derived value for all ^13^C chemical shifts in Figure 3 of *σ*_*ref*_  =  173 ppm was allowed for a better fit to experiment.^19^ The assignment was made against chemical shifts calculated from the geometry optimised (relaxed) FURSEM01 structure, and the fit is good and is compatible with a published ^1^H–^13^C CP‐HETCOR MAS NMR spectrum and GIPAW calculation by Widdifield et al.^9^ Note that in this J‐coupling‐based ^1^H–^13^C refocused INEPT experiment, we do not observe the H6 and H6' peaks found at 8.4 ppm for the dipolar‐coupling‐based ^1^H–^13^C CP‐HETCOR experiment in Widdifield et al.^9^ (Note that the purple contours observed in the ^1^H dimension at 6 and 10 ppm between 134 and 136 ppm in the ^13^C dimension are at the level of experimental noise.)

**Figure 5:**
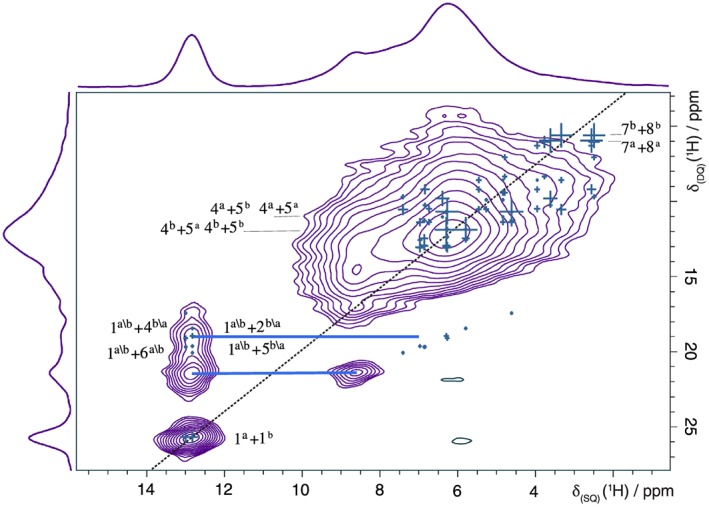
A ^1^H (500 MHz) DQ MAS (30 kHz) NMR spectrum, together with skyline projections, of furosemide from I, recorded using one rotor period of BABA recoupling. Sixty‐four transients were coadded for each of 256 t_1_ FIDs for a recycle delay of 3 s, corresponding to a total experimental time of 13.8 hr. The base contour level is at 7% of the maximum peak height. The F_1_  =  2F_2_ diagonal is shown as a dashed line. Horizontal lines (in light blue) indicate pairs of DQ peaks corresponding to close (<3.5 Å) H–H proximities. Crosses represent the GIPAW calculated shift with a dipolar coupling weighting. A partial assignment of the peaks is presented on the spectrum. The number refers to the label of the hydrogen (see Scheme 1) and the superscript to the molecular unit (a or b, corresponding to the two molecules in the asymmetric unit)

Figure 5 presents a ^1^H DQ MAS spectrum of furosemide form I recorded using fast MAS (30 kHz). The peaks correspond to pairs of protons with a significant (typically <3.5 Å)^51^ dipolar interaction, that is, they are close to each other in space. Experimentally, it is not possible to differentiate between intramolecular and intermolecular interactions. Using the MagresView software,^65^ the GIPAW calculated chemical shifts are represented as overlaid crosses, with a dipolar coupling weighting. The peaks are assigned to specific H–H pairs, and contributions both from intramolecular and intermolecular contributions are present.

### 3.2 Furosemide CSD deposited structures and polymorphs

Having considered a comparison of experimental NMR spectra and GIPAW calculations of form I of furosemide, we now use first‐principles calculations to assess the accuracy of the alternative XRD structures deposited for form I. We also examine the structures of the other two known forms of furosemide. Table 2 and 3 present the unit cell parameters and the dispersion‐corrected free energies of the geometry optimised structures, respectively. The three form I structures are close in energy—after a fixed cell optimisation, the three structures lie within 0.8 kJ mol^−1^; however, after a full relaxation, this is reduced to 0.1 kJ mol^−1^. Form I is known to be the experimentally most stable polymorph, and indeed, it has the lowest total energy, with forms II and III, 1.3 and 2.6 kJ mol^−1^ higher, respectively. Note that Nyman and Day showed that, for an analysis of over 500 organic molecular polymorphs, 95% of polymorphs are within 7.2 kJ mol^−1^.^66^

**Table 2:** Unit cell parameters and volume for fixed cell and relaxed cell geometry optimisations of furosemide CSD entries (see Table 1)

		Unit cell parameters						Volume per
		*a*	*b*	*c*	***α***	***β***	***γ***	molecule
		(Å)	(Å)	(Å)	(°)	(°)	(°)	(Å^3^)
F01	fixed	9.58	10.47	15.80	93.47	107.27	115.04	333.21
F01	relaxed	9.65	10.45	15.69	92.63	107.38	116.48	328.20
F03	fixed	9.59	10.50	15.71	93.06	107.22	116.21	331.21
F03	relaxed	9.65	10.45	15.61	92.65	107.33	116.57	328.80
F13	fixed	9.52	10.45	15.58	92.84	107.09	116.75	322.98
F13	relaxed	9.66	10.44	15.63	92.62	107.38	116.53	329.18
F14	fixed	5.01	10.11	26.62	90.00	95.40	90.00	335.52
F14	relaxed	5.05	10.03	26.55	90.00	94.38	90.00	335.04
F16	fixed	4.88	10.50	13.64	78.07	86.72	82.59	338.64
F16	relaxed	4.93	10.47	13.62	78.91	86.08	82.03	341.60

**Table 3:** Dispersion‐corrected final free energy differences (in kJ mol^−1^) between geometry optimised structures of furosemide (See Table 1)

	I			II	III
	F01^a^	F03	F13	F14	F16
Fixed	1.159	0.591	0.379	1.915	2.976
Relaxed	0.000	0.047	0.095	1.321	2.625

F01 relaxed is the reference structure.

NMR chemical shieldings have been calculated using the GIPAW method for all the geometry optimised furosemide structures. The full GIPAW calculated absolute chemical shielding for each distinct site in the asymmetric unit cells is presented in the Supporting Information.

The energetic similarity of the three form I structures is reflected in the root‐mean‐square deviation of atomic position. This is shown in Table 4. As expected, the differences between the fixed unit cell structures are greater than that between the relaxed unit cell structures. Table 5 summarises the differences in ^1^H magnetic shielding between the form I structures. The full GIPAW calculated magnetic shieldings for each distinct site in the asymmetric unit cells are presented in the Supporting Information. The maximal difference between the values is 0.2 ppm for structures after a fixed unit cell geometry optimisation. However, if we compare the structures that were geometry optimised under relaxed unit cell conditions, we see that the difference in shielding values minimise to a maximal difference of 0.08 ppm. This difference is comparable to the precision available in the solid‐state NMR experiments and much less than the typical discrepancy between GIPAW calculated and experimental chemical shifts. This shows us that, as far as NMR crystallography is concerned, each of the three form I structures (F01, F03, and F13) are an equally valid starting point, and the resulting relaxed unit cell structures are essentially indistinguishable.

**Table 4:** Root‐mean‐square deviation of atomic position (in Å) between geometry optimised structures of furosemide (See Table 1; from an overlay of 15 molecules, protons are included)

		F01	F03	F13	F01	F03	F13
		fixed	fixed	fixed	relaxed	relaxed	relaxed
F01	fixed						
F03	fixed	0.073					
F13	fixed	0.140	0.088				
F01	relaxed	0.106	0.076	0.103			
F03	relaxed	0.106	0.064	0.097	0.024		
F13	relaxed	0.104	0.061	0.102	0.040	0.021	

**Table 5:** Maximal difference in chemical GIPAW calculated shielding values and NICS and “H bond strength” (in ppm) for ^1^H nuclei (see Table 6; between geometry optimised form I structures: FURSEM01, FURSEM03, and FURSEM13

		Isolated	Full	Difference	NICS	H bond
		molecule	crystal	(Mol–Crys)		strength
Relaxed	mol a	0.06	0.08	0.07	0.07	0.06
	mol b	0.06	0.07	0.07	0.09	0.10
Fixed	mol a	0.09	0.20	0.19	0.13	0.19
	mol b	0.13	0.18	0.19	0.10	0.14

**Figure 6:**
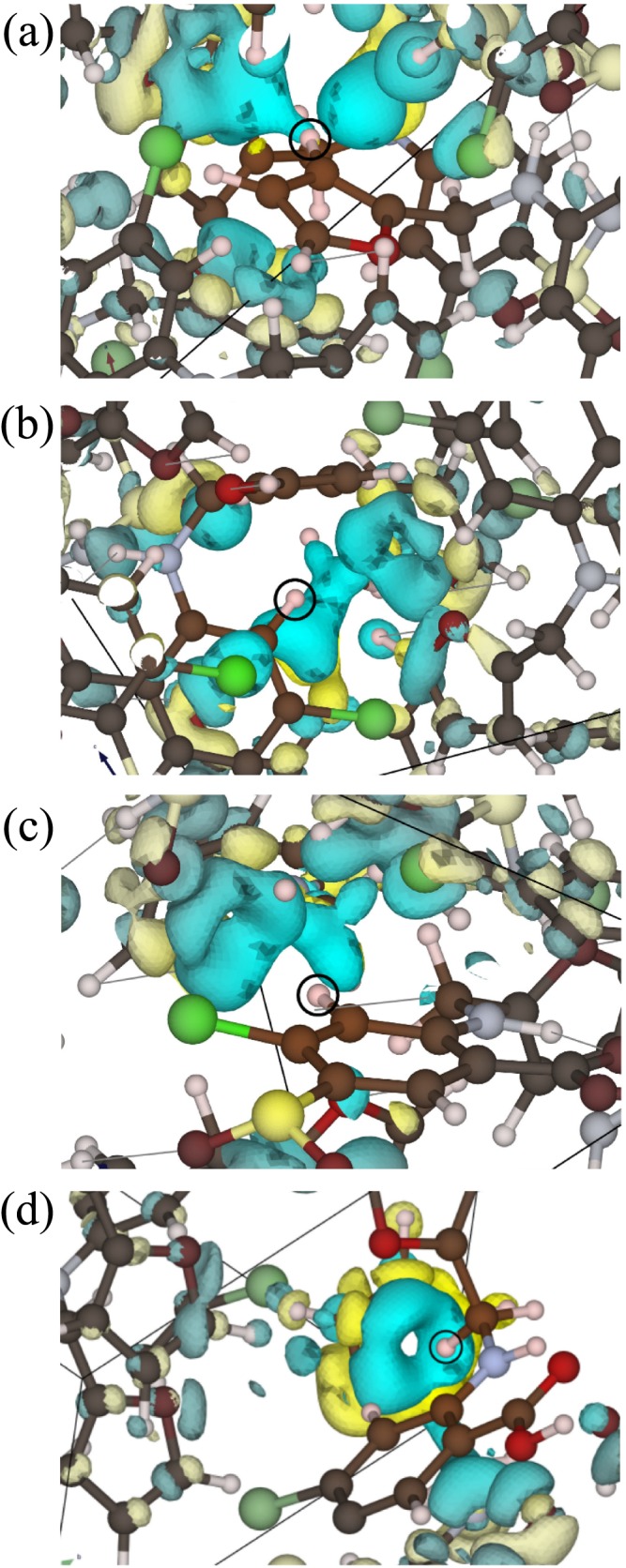
Decomposition maps^64^ visualizing the effect of aromatic ring currents on the NMR chemical shifts of (a) hydrogen 9 in molecule a in FURSEM01, (b) hydrogen 3 in molecule b in FURSEM01, (c) hydrogen 3 in FURSEM14, and (d) hydrogen 7 in FURSEM16

We now examine the changes in chemical shifts between the three polymorphs. A full listing of the GIPAW calculated chemical shifts values of the furosemide polymorphs, form I, II, and III, can be found in Table 6. When comparing different forms, the first component to examine is the calculated chemical shifts in an isolated molecule calculation. This is free from any effect due to intermolecular interactions. If the molecular conformations in the three forms are very similar, then the isolated molecule chemical shifts will be very similar. The only difference of >1 ppm exists for H8 between form III (F16) and the other configurations (1.68, 1.33, and 1.55 ppm difference with molecule a F01, molecule a F01, and F14, respectively). Between the three forms, the individual molecules display a variety of torsion angles about the N–C bond. However, the critical factor affecting the H8 ^1^H chemical shift appears to be the angle between the N–C bond and the furan ring. This is 93° in form III as compared with 111° and 114° for the other cases. The molecule to crystal changes in chemical shift encompass the effect due to intermolecular interactions and ring currents combined. This can be further decomposed into differences due to hydrogen bonds and to long‐range packing interactions. All forms have an OH...O hydrogen bond as the strongest intermolecular bond. In addition, at least one NH...X (where X is N or O) hydrogen bond exists in all forms, and forms I and II have two NH...X hydrogen bonds. The differences in longer range packing interaction are apparent in the calculated NICS values. In each polymorph, a different hydrogen interacts with an aromatic ring. The effect of the ring currents is visualised through Magnetic Shielding Contribution Field maps^64^) shown in Figure 6.

**Table 6:** GIPAW calculated chemical shifts^a^ and NICS (in ppm) for the hydrogen atoms in three furosemide polymorphs

			I			
			F01		II	III
			mol a	mol b	F14	F16
Isolated	*O* *H*	1	5.96	6.18	5.65	5.69
molecule	*N* *H*	2	7.64	8.09	7.95	8.44
	*C* *H*	3	5.76	5.32	5.26	5.91
	*N* *H*_2_	4	3.41	4.11	3.18	3.19
	*N* *H*_2_	5	4.09	3.50	3.92	4.49
	*C* *H*	6	7.38	7.52	7.52	7.60
	*C* *H*_2_	7	3.43	3.33	2.73	3.29
	*C* *H*_2_	8	2.86	3.21	2.99	4.54
	*C* *H*	9	5.29	5.46	5.29	5.54
	*C* *H*	10	5.42	5.44	5.52	5.49
	*C* *H*	11	6.64	6.69	6.72	6.63
Full	*O* *H*	1	13.46	13.63	12.76	12.92
crystal	*N* *H*	2	7.47	8.04	7.61	7.92
	*C* *H*	3	7.02	4.40	4.01	5.25
	*N* *H*_2_	4	5.24	6.92	6.47	6.08
	*N* *H*_2_	5	6.90	6.42	6.00	5.13
	*C* *H*	6	7.60	7.49	6.76	7.56
	*C* *H*_2_	7	4.25	3.97	2.95	1.83
	*C* *H*_2_	8	3.19	3.12	3.92	3.93
	*C* *H*	9	4.59	5.42	5.32	5.75
	*C* *H*	10	6.09	5.47	6.25	5.91
	*C* *H*	11	5.90	7.37	7.37	6.88
Difference	*O* *H*	1	7.50	7.45	7.12	7.22
(Crys–Mol)	*N* *H*	2	−0.17	−0.05	−0.34	−0.52
	*C* *H*	3	1.27	−0.92	−1.25	−0.66
	*N* *H*_2_	4	1.83	2.82	3.29	2.89
	*N* *H*_2_	5	2.81	2.92	2.08	0.63
	*C* *H*	6	0.23	−0.03	0.93	−0.04
	*C* *H*_2_	7	0.81	0.63	−0.76	−1.45
	*C* *H*_2_	8	0.33	−0.09	0.23	−0.61
	*C* *H*	9	−0.70	−0.04	0.65	0.20
	*C* *H*	10	0.67	0.03	0.73	0.42
	*C* *H*	11	−0.74	0.68	0.03	0.26
NICS	*O* *H*	1	1.32	1.91	1.95	1.58
	*N* *H*	2	0.14	−0.12	−0.04	0.17
	*C* *H*	3	−0.43	1.14	1.33	0.61
	*N* *H*_2_	4	−0.30	−0.21	−0.71	−0.23
	*N* *H*_2_	5	0.04	−0.47	−0.66	0.07
	*C* *H*	6	−0.13	−0.03	0.67	0.05
	*C* *H*_2_	7	−0.43	−0.06	0.23	1.53
	*C* *H*_2_	8	0.28	0.77	−0.32	0.41
	*C* *H*	9	1.52	0.92	0.03	−0.18
	*C* *H*	10	0.07	0.25	−0.56	−0.01
	*C* *H*	11	0.90	−0.18	0.23	−0.16
“H bond	*O* *H*	1	6.18	5.54	5.17	5.64
strength”	*N* *H*	2	−0.31	0.07	−0.30	−0.69
	*C* *H*	3	1.70	−2.06	−2.58	−1.27
	*N* *H*_2_	4	2.13	3.03	4.00	3.12
	*N* *H*_2_	5	2.77	3.39	2.75	0.56
	*C* *H*	6	0.35	0.00	−1.42	−0.09
	*C* *H*_2_	7	1.24	0.70	0.00	−2.98
	*C* *H*_2_	8	0.05	−0.86	1.25	−1.02
	*C* *H*	9	−2.22	−0.95	0.00	0.38
	*C* *H*	10	0.59	−0.22	1.29	0.43
	*C* *H*	11	−1.65	0.86	0.42	0.42

*Note*. NICS: nuclear independent chemical shift.

*σ*_*ref*_  =  29.60 ppm.

## 4 CONCLUSIONS

We have presented ^13^C CP MAS, ^1^H–^13^C refocused INEPT and ^1^H DQ MAS NMR spectra for form I of furosemide. The spectra have been assigned by comparison with chemical shifts computed using DFT and the GIPAW approach. We have used DFT calculations to assess the quality of three of the deposited structures for form I of furosemide. After a full relaxation of the atomic positions and unit cell parameters, all three structures are essentially indistinguishable, giving differences in GIPAW calculated ^1^H chemical shifts that are well below the precision obtained in our experiments. Further calculations on forms II and III of furosemide have employed a recently developed approach^64^ to identify short‐range and long‐range contributions to the chemical shift and further map the chemical origin of long‐range contributions. For the three polymorphs of furosemide, this approach highlights how the differing packing interactions influence the chemical shift, leading to observable differences in the NMR spectra. This suggests that such analysis will prove a useful tool in NMR crystallography. We note that this work has been used as an teaching example of NMR crystallography for workshops (see Supporting Information).

## Supporting information



Table S1: Shielding values (in ppm) of three geometry optimised form I structures ‐ relaxed structures, molecule 1
Table S2: Shielding values (in ppm) of three geometry optimised form I structures ‐ fixed structures, molecule 1
Table S3: Shielding values of three geometry optimised form I structures ‐ relaxed structures, molecule 2
Table S4: Shielding values of three geometry optimised form I structures ‐ fixed structures, molecule 2
Table S5: Difference in shielding values between relaxed and fixed geometry optimised structures of form II and I
