# Supplementary material for: An NMR crystallography investigation of furosemide
Source: Magn Reson Chem. 2018 Oct 11;57(5):191–9. doi: 10.1002/mrc.4789 (PMC6492277; doi:10.1002/mrc.4789)
Supplement: Supplementary file 1 — Table S1: Shielding values (in ppm) of three geometry optimised form I structures ‐ relaxed structures, molecule 1 Table S2: Shielding values (in ppm) of three geometry optimised form I structures ‐ fixed structures, molecule 1 Table S3: Shielding values of three geometry optimised form I structures ‐ relaxed structures, molecule 2 Table S4: Shielding values of three geometry optimised form I structures ‐ fixed structures, molecule 2 Table S5: Difference in shielding values between relaxed and fixed geometry optimised structures of form II and I [file MRC-57-191-s001.pdf]

An NMR Crystallography investigation of Furosemide - Supporting Information

Table S1: Shielding values (in ppm) of three geometry optimised form I structures - relaxed structures, molecule 1

			F01	F03	F13
Isolated Molecule	OH	1	23.64	23.66	23.67
	NH	2	21.96	21.95	21.94
	CH	3	23.84	23.83	23.84
	NH_2	4	26.19	26.18	26.18
	NH_2	5	25.51	25.53	25.54
	CH	6	22.22	22.23	22.23
	CH_2	7	26.17	26.16	26.17
	CH_2	8	26.74	26.73	26.73
	CH	9	24.31	24.30	24.30
	CH	10	24.18	24.15	24.15
	CH	11	22.96	22.90	22.90
Full Crystal	OH	1	16.14	16.16	16.18
	NH	2	22.13	22.14	22.13
	CH	3	22.58	22.58	22.61
	NH_2	4	24.36	24.33	24.32
	NH_2	5	22.70	22.73	22.75
	CH	6	22.00	22.02	22.03
	CH_2	7	25.35	25.38	25.35
	CH_2	8	26.41	26.43	26.42
	CH	9	25.01	25.00	24.99
	CH	10	23.51	23.55	23.55
	CH	11	23.70	23.67	23.62
difference (Mol - Crys)	OH	1	7.50	7.50	7.49
	NH	2	-0.17	-0.19	-0.19
	CH	3	1.27	1.26	1.23
	NH_2	4	1.83	1.85	1.85
	NH_2	5	2.81	2.80	2.78
	CH	6	0.23	0.21	0.20
	CH_2	7	0.81	0.79	0.81
	CH_2	8	0.33	0.30	0.31
	CH	9	-0.70	-0.71	-0.69
	CH	10	0.67	0.60	0.59
	CH	11	-0.74	-0.78	-0.73
NICS	OH	1	1.32	1.37	1.36
	NH	2	0.14	0.18	0.17
	CH	3	-0.43	-0.39	-0.38
	NH_2	4	-0.30	-0.27	-0.26
	NH_2	5	0.04	0.07	0.07
	CH	6	-0.13	-0.09	-0.09
	CH_2	7	-0.43	-0.38	-0.40
	CH_2	8	0.28	0.32	0.30
	CH	9	1.52	1.54	1.50
	CH	10	0.07	0.14	0.12
	CH	11	0.90	0.95	0.90
H Bond Strength	OH	1	8.82	8.87	8.85
	NH	2	-0.03	-0.02	-0.03
	CH	3	0.83	0.86	0.85
	NH_2	4	1.53	1.58	1.59
	NH_2	5	2.85	2.87	2.85
	CH	6	0.10	0.12	0.11
	CH_2	7	0.38	0.41	0.41
	CH_2	8	0.60	0.62	0.61
	CH	9	0.81	0.83	0.81
	CH	10	0.74	0.74	0.72
	CH	11	0.16	0.18	0.17

Table S2: Shielding values (in ppm) of three geometry optimised form I structures - fixed structures, molecule 1

			F01	F03	F13
Isolated Molecule	OH	1	23.64	23.67	23.64
	NH	2	22.17	22.08	22.15
	CH	3	23.85	23.83	23.83
	NH_2	4	26.18	26.18	26.19
	NH_2	5	25.52	25.53	25.52
	CH	6	22.17	22.19	22.18
	CH_2	7	26.16	26.17	26.16
	CH_2	8	26.74	26.72	26.75
	CH	9	24.30	24.29	24.30
	CH	10	24.17	24.14	24.16
	CH	11	22.95	22.89	22.90
Full Crystal	OH	1	16.10	16.26	16.17
	NH	2	22.24	22.20	22.26
	CH	3	22.75	22.69	22.59
	NH_2	4	24.24	24.37	24.35
	NH_2	5	22.90	22.79	22.76
	CH	6	21.93	21.99	21.98
	CH_2	7	25.33	25.38	25.35
	CH_2	8	26.33	26.38	26.46
	CH	9	25.04	24.98	25.00
	CH	10	23.71	23.64	23.50
	CH	11	23.68	23.65	23.67
difference (Mol - Crys)	OH	1	7.54	7.41	7.47
	NH	2	-0.08	-0.12	-0.11
	CH	3	1.10	1.14	1.24
	NH_2	4	1.94	1.81	1.84
	NH_2	5	2.62	2.74	2.76
	CH	6	0.24	0.20	0.20
	CH_2	7	0.83	0.79	0.81
	CH_2	8	0.41	0.33	0.28
	CH	9	-0.74	-0.69	-0.70
	CH	10	0.46	0.50	0.65
	CH	11	-0.73	-0.77	-0.78
NICS	OH	1	1.29	1.35	1.37
	NH	2	0.12	0.16	0.20
	CH	3	-0.41	-0.38	-0.39
	NH_2	4	-0.30	-0.26	-0.23
	NH_2	5	0.05	0.08	0.10
	CH	6	-0.13	-0.09	-0.09
	CH_2	7	-0.46	-0.39	-0.37
	CH_2	8	0.22	0.29	0.35
	CH	9	1.53	1.51	1.57
	CH	10	0.16	0.18	0.10
	CH	11	0.91	0.97	0.99
H Bond Strength	OH	1	8.83	8.76	8.84
	NH	2	0.04	0.04	0.09
	CH	3	0.69	0.76	0.84
	NH_2	4	1.64	1.55	1.61
	NH_2	5	2.67	2.82	2.86
	CH	6	0.11	0.11	0.11
	CH_2	7	0.36	0.41	0.45
	CH_2	8	0.63	0.63	0.63
	CH	9	0.79	0.81	0.86
	CH	10	0.62	0.69	0.75
	CH	11	0.18	0.20	0.21

Table S3: Shielding values of three geometry optimised form I structures - relaxed structures, molecule 2

			F01	F03	F13
Isolated Molecule	OH	1	23.42	23.48	23.48
	NH	2	21.51	21.54	21.52
	CH	3	24.28	24.29	24.29
	NH_2	4	25.49	25.48	25.49
	NH_2	5	26.10	26.09	26.09
	CH	6	22.08	22.09	22.10
	CH_2	7	26.27	26.28	26.28
	CH_2	8	26.39	26.40	26.40
	CH	9	24.14	24.13	24.12
	CH	10	24.16	24.12	24.12
	CH	11	22.91	22.88	22.88
Full Crystal	OH	1	15.97	15.96	15.96
	NH	2	21.56	21.58	21.58
	CH	3	25.20	25.20	25.20
	NH_2	4	22.68	22.65	22.72
	NH_2	5	23.18	23.24	23.22
	CH	6	22.11	22.11	22.13
	CH_2	7	25.63	25.63	25.67
	CH_2	8	26.48	26.50	26.52
	CH	9	24.18	24.16	24.14
	CH	10	24.13	24.12	24.09
	CH	11	22.23	22.23	22.22
difference (Mol - Crys)	OH	1	7.45	7.52	7.52
	NH	2	-0.05	-0.04	-0.06
	CH	3	-0.92	-0.91	-0.91
	NH_2	4	2.82	2.83	2.77
	NH_2	5	2.92	2.85	2.87
	CH	6	-0.03	-0.03	-0.03
	CH_2	7	0.63	0.65	0.62
	CH_2	8	-0.09	-0.10	-0.12
	CH	9	-0.04	-0.03	-0.02
	CH	10	0.03	0.00	0.03
	CH	11	0.68	0.66	0.66
NICS	OH	1	1.91	1.89	1.86
	NH	2	-0.12	-0.09	-0.10
	CH	3	1.14	1.17	1.15
	NH_2	4	-0.21	-0.12	-0.15
	NH_2	5	-0.47	-0.40	-0.42
	CH	6	-0.03	0.00	-0.02
	CH_2	7	-0.06	-0.03	-0.01
	CH_2	8	0.77	0.80	0.79
	CH	9	0.92	0.95	0.95
	CH	10	0.25	0.32	0.27
	CH	11	-0.18	-0.12	-0.14
H Bond Strength	OH	1	9.35	9.41	9.38
	NH	2	-0.17	-0.13	-0.16
	CH	3	0.22	0.26	0.23
	NH_2	4	2.61	2.70	2.62
	NH_2	5	2.46	2.44	2.45
	CH	6	-0.07	-0.03	-0.05
	CH_2	7	0.57	0.62	0.60
	CH_2	8	0.68	0.70	0.67
	CH	9	0.88	0.92	0.93
	CH	10	0.28	0.32	0.30
	CH	11	0.51	0.54	0.52

Table S4: Shielding values of three geometry optimised form I structures - fixed structures, molecule 2

			F01	F03	F13
Isolated Molecule	OH	1	23.41	23.49	23.48
	NH	2	21.65	21.63	21.52
	CH	3	24.30	24.29	24.29
	NH_2	4	25.49	25.48	25.49
	NH_2	5	26.10	26.08	26.09
	CH	6	22.04	22.07	22.10
	CH_2	7	26.26	26.29	26.28
	CH_2	8	26.37	26.40	26.40
	CH	9	24.13	24.11	24.12
	CH	10	24.15	24.12	24.12
	CH	11	22.91	22.89	22.88
Full Crystal	OH	1	15.93	16.06	15.96
	NH	2	21.66	21.64	21.58
	CH	3	25.14	25.12	25.20
	NH_2	4	22.73	22.68	22.72
	NH_2	5	23.13	23.31	23.22
	CH	6	22.07	22.09	22.13
	CH_2	7	25.53	25.61	25.67
	CH_2	8	26.50	26.48	26.52
	CH	9	24.26	24.24	24.14
	CH	10	24.08	24.06	24.09
	CH	11	22.32	22.23	22.22
difference (Mol - Crys)	OH	1	7.48	7.43	7.52
	NH	2	0.00	-0.01	-0.06
	CH	3	-0.85	-0.83	-0.91
	NH_2	4	2.76	2.80	2.77
	NH_2	5	2.96	2.77	2.87
	CH	6	-0.03	-0.02	-0.03
	CH_2	7	0.73	0.67	0.62
	CH_2	8	-0.13	-0.09	-0.12
	CH	9	-0.12	-0.12	-0.02
	CH	10	0.07	0.06	0.03
	CH	11	0.59	0.65	0.66
NICS	OH	1	1.85	1.82	1.86
	NH	2	-0.12	-0.10	-0.10
	CH	3	1.05	1.07	1.15
	NH_2	4	-0.18	-0.12	-0.15
	NH_2	5	-0.48	-0.43	-0.42
	CH	6	-0.04	-0.02	-0.02
	CH_2	7	-0.08	-0.03	-0.01
	CH_2	8	0.72	0.75	0.79
	CH	9	0.92	0.97	0.95
	CH	10	0.21	0.24	0.27
	CH	11	-0.13	-0.16	-0.14
H Bond Strength	OH	1	9.34	9.26	9.38
	NH	2	-0.13	-0.11	-0.16
	CH	3	0.20	0.24	0.23
	NH_2	4	2.58	2.68	2.62
	NH_2	5	2.49	2.34	2.45
	CH	6	-0.07	-0.04	-0.05
	CH_2	7	0.65	0.65	0.60
	CH_2	8	0.60	0.66	0.67
	CH	9	0.79	0.85	0.93
	CH	10	0.28	0.30	0.30
	CH	11	0.46	0.49	0.52

Table S5: Difference in shielding values between relaxed and fixed geometry optimised structures of form II and I

			Isolated Molecule	Full Crystal	difference (Mol - Crys)	NICS
	OH	1	0.033	0.291	-0.258	0.004
	NH	2	-0.061	-0.080	0.019	-0.004
	CH	3	0.000	-0.055	0.055	-0.063
	NH_2	4	0.005	0.228	-0.223	0.004
	NH_2	5	0.007	-0.039	0.046	0.012
F14	CH	6	0.014	0.023	-0.009	0.019
	CH_2	7	0.011	-0.083	0.095	-0.071
	CH_2	8	-0.009	-0.064	0.054	-0.108
	CH	9	-0.003	-0.018	0.015	-0.001
	CH	10	0.005	0.023	-0.018	0.002
	CH	11	-0.002	-0.060	0.058	-0.033
	NH		0.033	0.291	0.095	0.019
	OH	1	0.053	0.266	-0.213	-0.070
	NH	2	-0.025	-0.030	0.005	0.001
	CH	3	-0.015	0.002	-0.017	0.005
	NH_2	4	-0.022	-0.083	0.061	0.025
	NH_2	5	-0.019	-0.135	0.116	0.037
F16	CH	6	0.026	-0.044	0.070	0.005
	CH_2	7	0.010	0.077	-0.067	0.032
	CH_2	8	0.042	0.053	-0.011	0.011
	CH	9	-0.039	-0.002	-0.037	0.020
	CH	10	0.007	0.030	-0.023	0.016
	CH	11	0.005	0.034	-0.028	0.011
			0.053	0.266	0.116	0.037

Furosemide – NMR Crystallography analysis worksheet

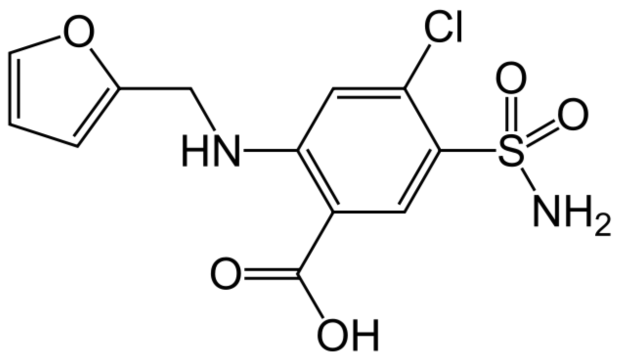

Furosemide is an organic molecule used in the pharmaceutical industry.

Furosemide has three polymorphs, for which structures have been deposited in the CSD.

PART I: Using Mercury software

You are presented with two .cif files, for form I (FURSEM01) and form III (FURSEM16).

(A, 10/100) Remembering the definitions of

Z (the number of molecules in the crystallographic unit cell) and Z' (the number of molecules in the asymmetric unit cell), complete the following:

Form I: Z' = _____ Z = ________

Form III: Z' = _____ Z = ________

(B_i, 10/100) For the form where Z' > 1, identify and state one difference in bond length (for directly bonded elements) between the distinct molecules within the asymmetric unit cell.

(B_ii, 10/100) For the form where Z' > 1, identify and state one difference (that is more than 0.5 Å) in a particular atom-atom distance between the distinct molecules within the asymmetric unit cell.

(C, 40/100) For both forms, identify the hydrogen bonding exhibited by (i) the COOH hydrogen and (ii) the NH_2 hydrogens.

Consider all X…Y distances up to 3.3 Å where the XHY bond angle is between 120° and 180°.

Present your analysis as a Table in the below format including a picture (prepared using Mercury) for each hydrogen bond, where you label the X…Y distance (in Å), the H…Y distance (in Å) and the XHY bond angle, and also include the atom labels.

Take care to ensure for the form where Z' > 1 to consider both distinct COOH protons and both NH_2 pairs of hydrogens. Also note that it is possible for a proton to hydrogen bond to more than one acceptor atom.

	d(X…Y) / Å	*d*(H…Y) / Å	Angle(XHY) / º
Form I			
O1-H1…O12	2.59	1.56	174.3

PART II: Using Magresview software

Read in the Furosemide Form I magres file (containing absolute chemical shieldings calculated using the density-functional theory (DFT) based GIPAW method, using the CASTEP software) into MagresView.

Look at the two solid-state NMR spectra provided.

(A, 20/100) **^{1}H- ^{13}C Refocused INEPT spectrum:**

Assign the peaks to the specific carbon atoms and enter the calculated isotropic absolute shielding, σ_{iso}, and the calculated isotropic chemical shift, δ_{iso}, using

$$\delta_{iso} = \sigma_{ref} - \sigma_{iso}$$

where

166.7 ppm for **Carbon (^{13}C)**

30.0 ppm for **Hydrogen (^{1}H)**

Note that the crosses in the spectrum denote the peak positions for the calculated isotropic chemical shifts.

**Peak label**	**Atom label**	**Carbon label**	**^{13}C Shielding / ppm**		**Hydrogen label**	**^{1}H Shielding /ppm**	
			**σ_{iso}**	**δ_{iso}**		**σ_{iso}**	**δ_{iso}**
A	5	C30	22.3	144.4	H16	22.5	7.6
B							
C							
D							
E							
F							
G							
H							

(B, 10/100) **^{1}H Double-Quantum (DQ) MAS spectrum:**

Assign the highest ppm ^{1}H peak. Prepare a picture using Mercury, showing the HH proximity (state also the distance) corresponding to the highlighted auto-correlation peak in the experimental spectra.
